# Epstein-Barr Virus (EBV)-associated Gastric Carcinoma

**DOI:** 10.3390/v4123420

**Published:** 2012-11-29

**Authors:** Hisashi Iizasa, Asuka Nanbo, Jun Nishikawa, Masahisa Jinushi, Hironori Yoshiyama

**Affiliations:** 1 Division of Stem Cell Biology, Institute for Genetic Medicine, Hokkaido University, N15 W7, Kita-ku, Sapporo 060-0815, Japan; Email:iizasah@igm.hokudai.ac.jp; 2 Graduate School of Pharmaceutical Sciences, Hokkaido University, N12 W6, Kita-ku, Sapporo 060-0812, Japan; Email:nanboa@pharm.hokudai.ac.jp; 3 Department of Gastroenterology and Hepatology, Yamaguchi University Graduate School of Medicine, Minami-Kogushi 1-1-1, Ube, Yamaguchi 755-8505, Japan; Email: junnis@yamaguchi-u.ac.jp; 4 Research Center for Infection-Associated Cancer, Institute for Genetic Medicine, Hokkaido University, N15 W7, Kita-ku, Sapporo 060-0815, Japan; Email:jinushi@igm.hokudai.ac.jp (J.M.); hironori@igm.hokudai.ac.jp (H.Y.)

**Keywords:** EBV, Carcinogenesis, EBV-associated gastric carcinoma, Epithelial, CD21, Methylation, miRNA

## Abstract

The ubiquitous Epstein-Barr virus (EBV) is associated with several human tumors, which include lymphoid and epithelial malignancies. It is known that EBV persistently infects the memory B cell pool of healthy individuals by activating growth and survival signaling pathways that can contribute to B cell lymphomagenesis. Although the monoclonal proliferation of EBV-infected cells can be observed in epithelial tumors, such as nasopharyngeal carcinoma and EBV-associated gastric carcinoma, the precise role of EBV in the carcinogenic progress is not fully understood. This review features characteristics and current understanding of EBV-associated gastric carcinoma. EBV-associated gastric carcinoma comprises almost 10% of all gastric carcinoma cases and expresses restricted EBV latent genes (Latency I). Firstly, definition, epidemiology, and clinical features are discussed. Then, the route of infection and carcinogenic role of viral genes are presented. Of particular interest, the association with frequent genomic CpG methylation and role of miRNA for carcinogenesis are topically discussed. Finally, the possibility of therapies targeting EBV-associated gastric carcinoma is proposed.

## 1. Introduction

Epstein-Barr virus (EBV) is a ubiquitous human herpes virus with oncogenic activity. The EBV genome can be detected in malignancies of both lymphoid and epithelial cell origin, such as Burkitt’s lymphoma (BL) and nasopharyngeal carcinoma (NPC) [1,2]. In 1990, EBV genomes were detected in gastric carcinomas using polymerase chain reaction [3] and *in situ* hybridization (ISH) for EBV-encoded small ribonucleic acid 1 (EBER1). These findings indicated that EBV-associated gastric carcinomas (EBVaGC) comprise about 10% of all gastric carcinomas worldwide [4,5,6]. Since EBVaGC are monoclonal proliferations of a single cell persistently infected with EBV, EBV infection may be involved in the early stages of gastric carcinogenesis [7,8,9].

EBV spreads by the oral route [10]. After primary infection, EBV establishes the lifelong virus carrier state, called latent infection, which expresses a limited set of viral genes required for viral episome maintenance, thereby conferring a survival advantage to the infected cell. BL, approximately half of the NPC, and EBVaGC belong to latency I, in which EBV nuclear antigen 1 (EBNA1), EBER1 and 2, and *Bam*HI-A rightward transcripts (BART) are expressed. Latency II neoplasm includes the remaining NPC and Hodgkin’s lymphoma (HL) and is characterized by the expression of latent membrane protein 1 (LMP1) in addition to latency I transcripts. Latency III neoplasms, typified by lymphomas observed in immunosuppressed patients, additionally expresses EBNA2, 3A, 3B, 3C, and LP. Three promoters (Cp, Wp, and Qp) are utilized for EBNA transcription. The Cp- or Wp-initiated large transcript is differentially spliced into six EBNA mRNAs as observed in latency III cells, whereas Qp mediates selective expression of EBNA1 in Latency I or II cells [7]. The expression of latent genes is under a strict epigenetic regulation through DNA methylation. Cp and Wp are hypermethylated in latency I [11].

EBVaGC is a latency I neoplasm and expresses EBNA1, EBER, BART, and sometimes (40%) latent membrane protein 2A (LMP2A) [9]. Since EBVaGC does not express EBNA2 and LMP1, which are important for B cell immortalization and transformation [2,7], preexisting abnormalities may exist in gastric epithelial cells [12]. Not only viral gene, but also the host cell DNA methylation has been frequently observed in EBVaGC [13,14,15]. Promoter hypermethylation of tumor-related genes is known to cause down-regulation of their gene expression [16]. Target gene silencing by viral micro RNAs (miRNAs) [17] has also been observed in EBV infected cells. Both mechanisms may influence the tumor progression of EBVaGC.

## 2. Definition, Epidemiology, and Clinical Features

### 2.1. Definition

**Figure 1:**
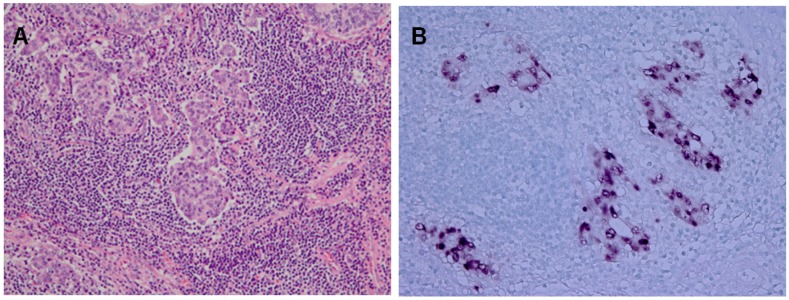
Lymphoepithelioma-like subtype of Epstein-Barr virus-associated gastric carcinomas (EBVaGC). **A.** Hematoxylin-Eosin Staining. **B.**EBV-encoded small ribonucleic acid-*in situ* hybridization (EBER1-ISH) demonstrates positive nuclei in the carcinoma cells, which are surrounded by infiltrating lymphocytes.

Almost 10% of the gastric carcinomas throughout the world are monoclonal proliferations of EBV-carrying tumor cells [4]. A characteristic feature of EBVaGC is lymphoepithelioma-like carcinoma, which presents a diffuse-type histology with lymphoid infiltration. EBVaGC is defined by the presence of EBV in neoplastic cells. EBER1-*in situ* hybridization (ISH) is used to identify EBVaGC, because EBER1 is highly abundant (10 million copies per cell) in each infected cell. Typically, tumor cells, of which nuclei are positive for EBER1-ISH, are surrounded by lymphoid stroma (Figure 1). EBVaGC has distinct clinicopathological features, which predominantly arises in men and presents a generally diffuse histological type [18].

### 2.2. Epidemiology

Most studies did not show evident age dependence of EBVaGC frequency. Almost all studies have shown male predominance of EBVaGC, suggesting that risk from lifestyle or occupational factors may exist among males [19]. An interview study in Japan showed that salty food intake and wood dust and/or iron filings exposure, which may induce mechanical injury to the gastric epithelia, are related to a higher EBVaGC risk [20].

In contrast to BL and NPC, which are endemic in Equatorial Africa and Southeast Asia, respectively, EBVaGC is a non-endemic disease distributed throughout the world [6]. However, there are some regional differences in the incidence of EBVaGC. The incidence of EBVaGC in all cases of gastric cancer is distributed from highest (16-18%) in the USA and Germany to the lowest (4.3%) in China [6,21,22]. A Japanese study investigated incidence of EBV-positive cases in all gastric cancers in several areas. The study indicated that EBVaGC prevalence was inversely related to the GC incidence [23]. Prognosis of EBVaGC is relatively favorable.

### 2.3. Clinical Features

**Figure 2:**
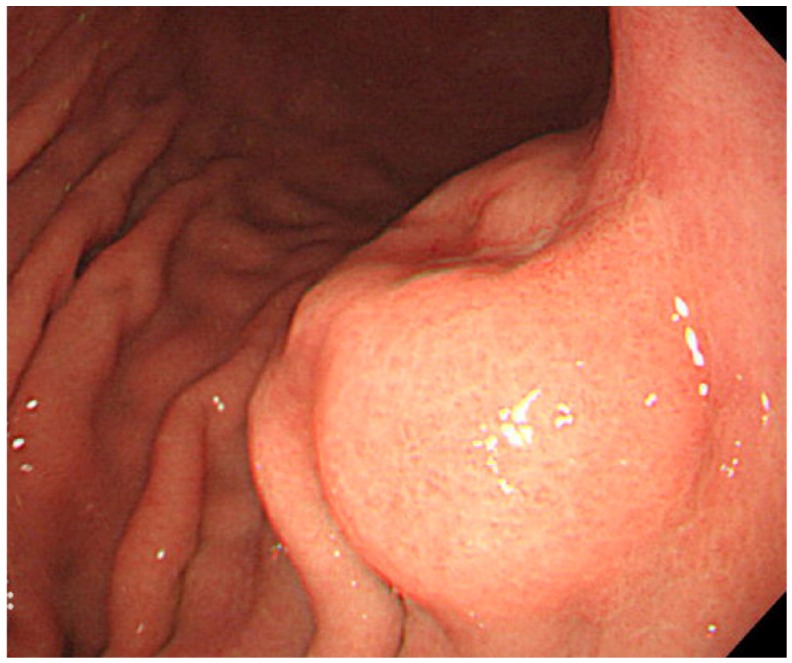
Endoscopic image of an early EBVaGC in the upper gastric body. The tumor shows protruded shape probably because of the abundant lymphocyte infiltration.

The most useful modality for the diagnosis of gastric carcinoma is endoscopy. By endoscopy, EBVaGC appears as superficial depressed (or ulcerated) lesions in the upper part of the stomach (Figure 2). Tumor locates predominantly in the non-antrum part of the stomach [19]. Because gastric cancer related to *Helicobacter pylori* (Hp), a causative agent of chronic gastritis, intestinal metaplasia, and cancer, locates predominantly in the antrum, these pathogens have been thought to cause gastric cancer by independent mechanisms [19]. Gastritis related to Hp frequently starts in the antrum. However, Yanai *et al.* reported that EBVaGC are frequently located near the mucosal atrophic border, where mild to moderate chronic atrophic gastritis (CAG) is common [24]. They also showed frequent detection of both EBV and Hp at the mucosa with moderate CAG, where inflammatory cell infiltration is abundant, and not at the mucosa with marked CAG, where inflammatory cell infiltration is scarce [25].

## 3. Route of Epithelial Infection

EBV infects human B lymphocytes and epithelial cells via different entry mechanisms. In case of B cells, the major outer envelope glycoprotein, gp350/220, is responsible for attachment of the virus with high affinity to CD21 or the human complement receptor type 2 (CR2) on B cell surface [26,27,28,29]. EBV is subsequently internalized into the cells via endocytic pathway. Fusion with viral envelope and endosomal membrane of B cells is triggered by the interaction of a second envelope glycoprotein, gp42, with HLA class II [30], and is thereafter mediated by the core fusion complex, gH/gL/gp42 [31,32].

In contrast, the mechanism by which EBV infects human epithelial cells remains unclear. Human epithelial cells are CD21-negative or express CD21 at low level in some epithelial cells in culture and highly resistant to cell-free EBV infection [33,34]. At least three models have been proposed as mechanisms for the EBV attachment to epithelial cells independent of CD21. First, it has been demonstrated that EBV virions coated with immunoglobulin A (IgA) specific to gp350/220 can bind efficiently to the polymeric IgA receptor [35]. Polymeric IgA is commonly present in human saliva and binds to the secretory component (SC) protein, which is a transmembrane protein expressing on the basolateral surfaces of polarized epithelial cells. A complex of EBV/IgA/SC is internalized into epithelial cells via endocytic pathway. This may be relevant to infection through the basolateral surface of an epithelial cell, which presumably resembles the physiological environment that the virus encounters *in vivo* [36]. Second, a complex of gH and gL was proposed to serve as epithelial ligands in the absence of CD21. EBV derived from B cells binds with high affinity to CD21-negative epithelial cells, but recombinant viruses lacking gH/gL lose this ability [31,32], suggesting that there is an epithelial cell-specific receptor for gH/gL that serves in attachment of EBV. It has been also shown that the direct interaction between gH/gL and the integrins αVß6 and αVß8 can provide the trigger for fusion of EBV and plasma membrane of epithelial cells [37]. Finally, an interaction between an EBV-encoded membrane protein, BMRF2, and integrins on polarized epithelial cells has been proposed as a model for EBV attachment to the cell surface [38]. The tripeptide Arg-Gly-Asp (RGD) motif in the BMRF2 molecule is presented as a ligand for ß1, α5, α3, and αV integrins [39,40]. However, BMRF2 is not required for cell-to-cell fusion [41,42] and apparently very few BMRF2 molecules exist in the virion [43]. It remains unclear whether the interaction of BMRF2 with integrins is primarily responsible for attachment and/or post-attachment events.

Interestingly, EBV virions released from B cells are deficient in gp42 which renders them more efficient to infect epithelial cells, but less efficient to infect B cells [44,45]. In contrast, EBV released from infected epithelial cells possesses gp42 and efficiently infects B cells [44]. This change in cell type tropism for EBV infection suggests that EBV shuttles between epithelial cells and B cells in the host during infection cycle. This observation supports a model that pharyngeal epithelial cells are in lytic EBV infection and shed infectious EBV particles for transmission.

The fusion of EBV envelope with the plasma membrane of epithelial cells requires a trigger gH/gL complex [36,45,46,47,48,49]. The fusion of EBV with an epithelial cell is likely triggered by a direct interaction between gH/gL and unknown epithelial cell surface molecules, which might be the identical proteins that can serve as attachment receptors in the absence of CD21 [50].

**Figure 3:**
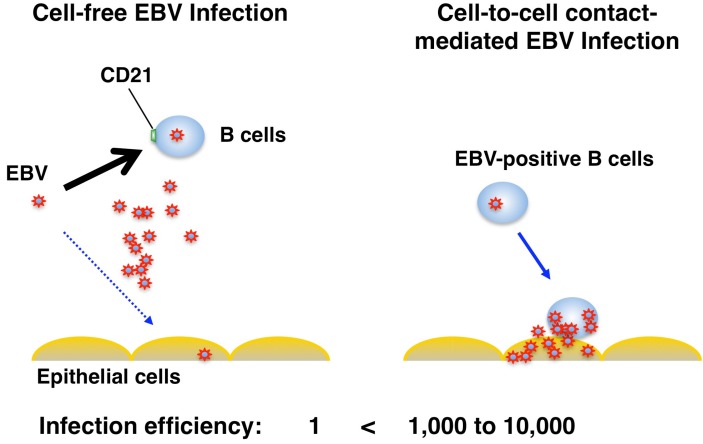
Cell-to-cell contact-mediated EBV transmission to epithelial cells. In cell-free infection, EBV preferentially infects B cells using CD21 receptor. EBV also infects CD21-negative epithelial cells as part of its normal life cycle, however much less efficiently. EBV transfer mediated by cell-to-cell contact with B cells increases the infection efficiency 1,000 to 10,000-fold compared with cell-free virus infection.

Several lines of evidence indicate that EBV infection into epithelial cells is mainly mediated by cell-to-cell contact [34,51,52,53,54,55]. The rate of EBV infection in epithelial cells is 10^3^-fold higher by co-culturing with EBV-positive B cells than by cell-free infection [34,51,55]. Moreover, most EBV virions are retained on cell surfaces after binding to primary B cells and transferred to epithelial cells, resulting in the 10^3^ to 10^4^-fold increase of infection compared with cell-free virus infection [53,54]. All these studies support a model that EBV-infected B cells migrating into the epithelial stroma or intraepithelial space contribute to the efficient EBV transmission into epithelium via cell-to-cell contact (Figure 3). The detailed molecular mechanisms of cell-to-cell EBV transmission remain unclear. Shannon-Lowe *et al.* showed that EBV virions loaded on the surface of primary B cells facilitate the formation of a virological synapse (VS)-like intercellular conjugation between B cells and co-cultured epithelial cells [53,54]. The VS is a tight adhesive junction across which virus can be efficiently transferred from virus-infected cells to non-infected target cells without cell-cell fusion [56]. An important role of an EBV glycoprotein, BMRF2 in the cell-to-cell spread of EBV in polarized oral epithelial cells has been proposed [39,40]. The mucosa of oropharyngeal and nasopharyngeal regions are known to be heavily filtrated by lymphocytes, suggesting that cell-free virus in the saliva could first bind to the surface of B cells and then efficiently transfer to pharyngeal epithelial cells through a cellular conjugate between B cells and epithelial cells.

The detailed molecular mechanism by which EBV infects epithelial cells still remains unclear. Some key factors involved in viral attachment, membrane fusion, and cell-to-cell contact-mediated viral transmission, have been identified [48,49]. However, no cellular receptors for EBV infection have yet been identified on epithelial cells. Also very little insights have been provided for a conceptual understanding of viral entry mechanism into epithelial cells. Further investigations are still required.

## 4. Viral Genes and Carcinogenesis

### 4.1. Models of EBV infection of gastric epithelial cells

EBV infects both B lymphocytes and epithelial cells, since the virus is discovered in BL cells, HL cells, NPC cells, and EBVaGC cells. Experimental EBV infection to B cells is very efficient, since EBV uses high affinity receptor, CD21 for its entry [16]. However, epithelial cells are CD21-negative and infection of epithelial cells could not be achieved for a long time, exceptionally when CD21 expression was overcome by gene transfer [33,57].

We have clearly proved direct infection of human gastric epithelial cells by EBV [50]. The infection was achieved by using a recombinant EBV with a selectable marker gene [58,59], but without any operations, such as introduction of the CD21 gene. In our study, epithelial cells were negative for CD21 and the infection was not blocked by anti-CD21 monoclonal antibody [50]. We have next showed that efficient transfer of EBV to epithelial cells by mixed culture with recombinant EBV producing B cells [34]. Other than experimentally EBV-infected cells, SNU-719 cell [60] and KT cell [61] are few cells retaining the same clonal EBV genome and EBV gene expression pattern of latency I as the original tumor biopsy. EBV-harboring epithelial cells are difficult to propagate *in vitro* and in animal models.

### 4.2. Growth promoting effects of EBV

The KT cell is a good *in vivo* model of EBVaGC and expresses high IL-1ß compared with EBV-negative gastric tumor cells [62]. Primary cell cultures from healthy gastric mucosal biopsies were infected with recombinant EBV [63]. The established cells expressed Qp-driven EBNA 1, EBER, BART, and LMP2A, similar to EBVaGC. The EBV-positive clones showed rapid proliferation and p53 overexpression, and exhibited anchorage independence in colony formation assay.

Growth promotion by EBV infection was also observed in EBV-infected NU-GC-3 cells through secretion of insulin-like growth factor (IGF)-1 as an autocrine growth factor [64]. It has been shown that EBERs play an oncogenic role by inhibition of apoptosis [65,66] and IGF-1 induction [67]. The oncogenic role of other genes, such as BARF1 [68] and LMP2A [69] has been reported. A recent report showed that EBV infection affected miRNA expression [70]. While no consensus exits as to the exact mechanism by which EBV promotes EBVaGC, the establishment of EBVaGC cell lines and the development of an *in vitro* model represent significant progress towards this goal.

## 5. Virus and Host Interactions at Molecular Level

### 5.1. DNA Hypermethylation in EBV and Host Genomes

A number of CpG islands in the promoter region of an tumor suppressor gene have been methylated in cancer cells than in normal cells [71]. Expression of many genes, such as *p16* and *RUNX3*, is suppressed in stomach cancer owing to promoter methylation [72,73]. Promoter hypermethylation is especially frequent in EBVaGC [74,75,76]. Methylation of promoter region in *APC*, *p16*, *MINT1*, *MLH1*, *TP73*, and *HOXA10* [14,77,78] has been specifically observed in EBVaGC (Table 1). A large-scale analysis revealed that *CXXC4*, *TIMP2*, and *PLXND1* are specifically methylated in EBVaGC [79]. Down regulation of CXXC4, a suppressor of the Wnt pathway, promotes tumor cell proliferation and invasiveness [80]. Down regulation of TIMP2, a suppressor of metalloproteinase, inhibits tumor cell metastasis [81]. Methylation of similar genes has been reported in cancers associated with hepatitis B or C infection [82,83], suggesting that a common mechanism may underlie the formation of infection-associated cancers. 

**Table 1:** DNA hypermethylation in EBVaGC.

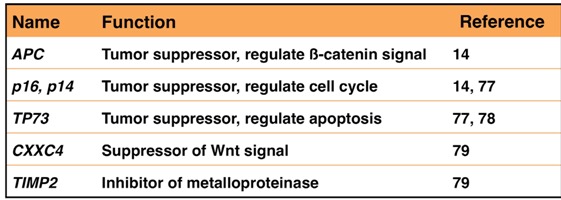

However, the precise molecular mechanism of host DNA methylation during the early stage of EBV infection of the gastric epithelium is not fully understood. It is reported that LMP2A induces the phosphorylation of STAT3, which activates DNA methyltransferase 1 (DNMT1) transcription and causes PTEN expression loss through CpG island methylation of the *PTEN* promoter [69]. Although LMP2A is expressed in substantial cases of EBVaGC [84], EBVaGC patients are usually negative for LMP2A antibody [85]. Constitutive overexpression of DNMT1 has been observed in EBV-infected gastric epithelial cells that do not express LMP2A significantly [69]. Further investigations using EBV mutants of LMP2A [86] may uncover its precise molecular mechanism.

### 5.2. miRNA and Carcinogenesis

miRNAs are endogenous 18 - 25 nt RNAs and play important gene-regulatory roles in eukaryotic cells via posttranscriptional repression of gene expression. miRNA targets 3′-untranslated region (UTR) elements of mRNA and mediates mRNA decay through degradation of polyA (RNA silencing: RNAi) [87]. Different expression patterns of miRNA from normal tissues were expected in cancer. Several miRNAs and non-coding RNAs have been found to have links with some types of cancer and are referred as “oncomirs”. This is because miRNAs have a role as oncogenes when they target tumor suppressor genes. On the other hand, miRNAs are tumor suppressors when they target oncogenes [88]. 

A clonal EBV infection has been found in EBV-associated epithelial tumors, such as NPC and EBVaGC [9,89]. EBV encodes a large number of miRNAs [90]. Up to 25 pre-miRNAs are encoded in the BHRF1 and BART regions of the genome, which result in four mature BHRF1 miRNAs and 40 BART miRNAs [91]. A prototypic EBV strain B95-8 is known to have 11 kbp deletion in BART region. However, B95-8 virus can be produced in large amount and possesses prominent B cell transformation ability. Moreover, a recombinant virus lacking entire region of BART region was still able to infect and transform B cells [92]. These findings indicate that BART transcripts are not required for B cell transformation and neglected the importance of BART for a long period. Cai *et al.* identified 13 *BART* miRNAs in the region of B95-8 deletion [91].

**Table 2:** EBV-derived miRNAs and their target genes.

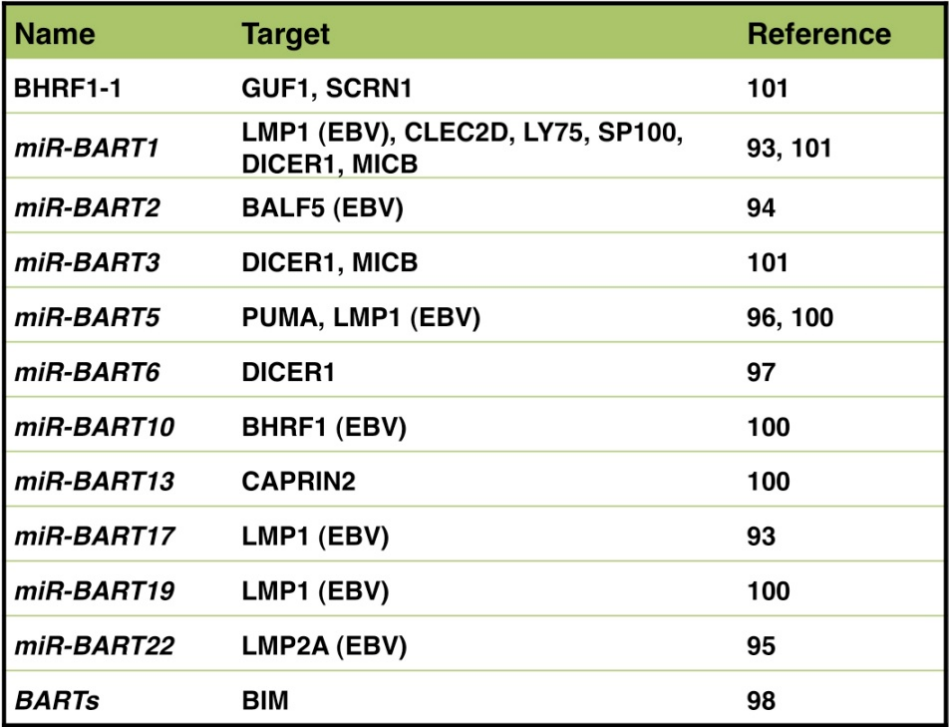

EBV-encoded BART miRNAs target the 3'-UTRs of viral genes, such as LMP1, BALF5, and LMP2A genes, and negatively regulate expression of these viral genes [93,94,95]. On the other hand, EBV miRNAs repress cellular proteins, which include p53 up-regulated modulator of apoptosis (PUMA), DICER1, and BIM [96,97,98] (Table 2). Preservability of the 3'-UTR domain of the target gene between species is a common feature of mammalian miRNA. However, since EBV preferentially infects humans, some EBV miRNAs characteristically target only human gene and not genes for other mammals. EBV miRNAs have another characteristic feature, some of which binding motifs are found only on EBV genome and not preserved on other viral genomes [97,99]. In order to identify EBV miRNA targets, a transcriptome-wide identification of miRNA binding sites has been performed between EBV-negative and EBV-positive cell lines [99,100,101].

Biological significance of viral miRNA in EBV-infected cells was searched using EBV recombinants. In these recombinants, mutations were systematically introduced in EBV’s precursor miRNA transcripts to prevent their subsequent processing into mature viral miRNAs. Phenotypic analyses of miRNA mutants revealed that the viral miRNAs contribute to EBV-associated cellular transformation rather than regulation of viral lytic replication [102].

There are two complicated stories in EBV-infected cells. One is that expression of viral latent genes induces cellular miRNAs. Viral LMP1 induces human miR-146a and miR-155 expression [103,104,105]. And excessive miR-155 expression is known to form B lymphoma [106]. Two, infection of EBV conversely suppresses entire host cell miRNA expression [70,107]. Since down-regulation of the cellular microRNA family miR-200 causes epithelial-mesenchymal transition (EMT), this phenomenon must be an important step in the process of malignant transformation of both EBVaGC and nasopharyngeal carcinoma cells. The precise mechanism for dysregulation of host miRNA by EBV infection needs to be clarified. It is known that low expression level of miRNA processing enzymes, DICER1 and DROSHA, is well correlated with tumor progression in many cancers [108]. We have shown that the expression level of human DICER1 is also lower in EBV-infected cells than in non-infected cells [97,101]. These findings suggest that progression of EBV-associated tumor is possibly regulated by EBV through regulation of viral and host miRNA expression. However, most of the studies presented in this section are performed using BL cells. Further study using EBV positive gastric epithelial cells is required.

## 6. Diagnosis and Treatment of EBV-associated GC

### 6.1. Procedure

EBER1-ISH is a most sensitive method to identify EBV infection. Application of EBER1-ISH to gastric mucosal biopsy samples from patients who have undergone upper gastrointestinal endoscopy is very useful to make diagnosis of EBVaGC before treatment. Patients with EBVaGC had elevated serum antibodies against EBV early antigen and EBV capsid antigen. However, EBNA1 antibody titers did not show significant difference between patients and healthy counterparts [9]. 

**Figure 4:**
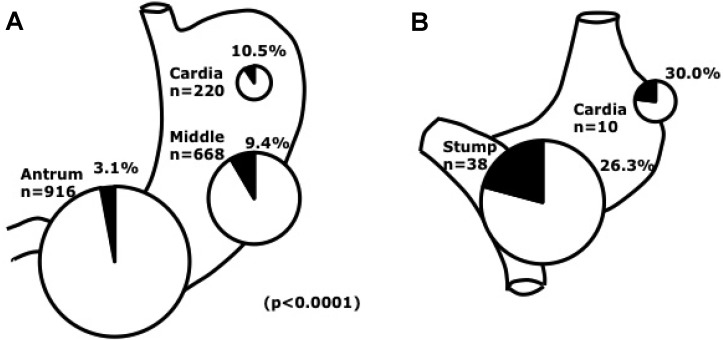
EBVaGC by site in the stomach. **A.** Distribution of EBVaGC. EBV prevalence was more frequent in the cardia and middle stomach than in the antrum, where over half of EBV-negative gastric cancers were located. **B.** EBV involvement in remnant cancer by site. Note that frequency of EBV infection in the non remnant cardiac cancer was 10.5% [22].

Yanai *et al.* examined 124 gastric carcinomas from 117 patients using EBER1-ISH. Among them, twelve tumors (9.7%) were identified as EBV associated [109]. An interesting feature of EBVaGC is its predominance in the non-antrum part of the stomach (Figure 4A). EBVaGC appears as a superficial depressed- or ulcerated-lesion in the upper part of the stomach. Histology of EBVaGC is mainly diffuse-type carcinoma rich in lymphocyte infiltration (gastric carcinoma with lymphoid stroma). Endoscopic ultrasonography revealed a hypoechoic mass in the third layer, reflecting submucosal nodules [110]. 

Gastric remnant cancer (gastric stump cancer) after distal gastric resection for benign disease, such as refractory gastric or duodenal ulcer disease or recurrent ulcer with gastric outlet obstruction, remains a substantial clinical concern, because the incidence of remnant cancer is still increasing (Figure 4B) [111]. A high prevalence of EBV involvement (25 to 41.2%) in gastric remnant carcinoma has been reported. High cell proliferation activity in the epithelium has been reported. The reflux of bile and pancreatic juice is considered to cause regenerative atypia and cell proliferation in epithelial cells [112]. Atrophic change of remnant gastritis in Billroth-II anastomoses was associated with EBV-positive gastric remnant carcinoma with high incidence [113].

### 6.2. Prognosis

To date there is no specific therapeutic method for EBVaGC. Since the frequency of undifferentiated type of cancer is high in EBVaGC, most of the tumor is surgically resected. CpG island methylation of the promoters of various tumor-related genes plays important roles in the development and progression of gastric cancer [16]. Statistical analysis showed that promoter hypermethylation is more frequently observed in EBVaGC than in EBV-uninfected gastric carcinoma [69,74,75,76]. Medical treatment with a demethylation agent, which induces lytic EBV infection in latently EBV infected cells, may lead to a lysis of cancer cells. This approach could be applied to the medical treatment of EBVaGC, since methylation of the tumor suppressor gene is also a key abnormality in EBVaGC [12,114,115].

Most of the previous reports did not observe any prognostic difference between EBV-positive and -negative gastric cancer [21]. However, early EBVaGC has the low frequency of lymph node metastasis even in submucosal type. Partial medical treatment, such as endoscopic treatment, can be adapted to such a case. The authors have experienced a case of early EBVaGC with submucosal invasion, in which palliative endoscopic treatment was performed. Recurrence was not observed in this case for more than four years [116]. A clinicopathological study in the Netherlands mentioned that EBVaGC accompanied lymph node metastasis in significantly lower frequency than EBV-negative stomach cancer. In the study, EBVaGC cases showed a better prognosis than negative cases [117]. 

## 7. Conclusion

Considerable studies suggest that EBV contributes to cell proliferation and survival, and may directly contribute to the development of EBVaGC through these effects. EBV affects multiple host proteins and pathways that normally promote apoptosis and regulate cell proliferation. However, the underlining molecular mechanisms of these effects are complex and certainly affecting each other. 

In another aspect, inflammation of the stomach will recruit EBV-infected B-lymphocytes in the vicinity of gastric epithelia and may increase the frequency of EBV infection of epithelia. Differences in individual inflammatory response by either genetic and/or environmental effect, such as a predisposing loss of ARID1A in epithelial cells before EBV infection [118] or single nucleotide polymorphisms of promoter region of interleukin-10 and/or tumor necrosis factor-α [119], possibly affect the oncogenic pathway to EBVaGC.
